# The Fabrication of Fragrance Microcapsules and Their Sustained and Broken Release Behavior

**DOI:** 10.3390/ma12030393

**Published:** 2019-01-27

**Authors:** Hongbin Zhao, Xuening Fei, Lingyun Cao, Baolian Zhang, Xin Liu

**Affiliations:** 1 School of Chemical Engineering and Technology, Tianjin University, Tianjin 300072, China; zhaohongbintj@126.com; 2 School of Science, Tianjin Chengjian University, Tianjin 300384, China; 3 School of Materials Science and Engineering, Tianjin Chengjian University, Tianjin 300384, China; ybysw@126.com; 4 Tianjin AnYing Bioengineering technology Co., Ltd., Tianjin 300384, China; 13502014135@163.com

**Keywords:** microcapsule, sustained release, broken release, control release, SPME-GC-MS, fragrance, textile finishing, melamine-formaldehyde

## Abstract

Their controlled release property is the most important feature of functional microcapsules and carriers. In this work, melamine resin shell fragrance microcapsules were fabricated in a non-ionic system, and their chemical structure, particle size, and morphology were analyzed. The sustained release property of the prepared microcapsules over 2400 h was studied with a weighing calculation method, and based on the fitting results, the release rate trend was consistent with the Peppas model (y = 100 − 2.30t^0.3213^). Furthermore, the sustained and broken release behavior of the microcapsules in impregnated fabric samples were investigated for the first time by our proposed Solid Phase Microextraction-Gas Chromatography-Mass Spectrometer (SPME-GC-MS) method. The qualitative and quantitative analysis results showed that the middle and base note compositions were outstanding in the sustained release state, and the top note showed more advantages in the broken release state. In addition, it was found that the characteristic peak species and intensities of the sample finished with the microcapsules were more similar to pure essence oil than the sample finished by traditional methods, suggesting that the prepared microcapsules showed an excellent odor recovery and strength.

## 1. Introduction

Microencapsulation is a technology that encapsulates active and volatile substances to form nano- or micro-scale capsules. It may be able to protect the core materials from the surrounding environment and provide some new applications and release characteristics. Based on these excellent characteristics, microencapsulation has been widely applied in the fields of perfume [1], antimicrobial materials [2], medical materials [3,4], phase-change materials [5], self-healing materials [6], damage sensing [7], flame retardants [8], magnetic materials [9], lubricants [10], and explosives [11], and it has developed a series of valuable products. 

In the application of microencapsulation, the controlled-release property of the core material is an important evaluation indicator [12]. However, the detection and evaluation of core material release from the microcapsules is a difficult problem, and it has been a research hotspot for decades. Up to now, many methods such as spectroscopy, chromatography, and thermal analysis method have been applied to study the application of microencapsulation based on the properties of core materials. Generally, the core materials are volatile substance, and they can be detected by the chromatography. For example, the Gas Chromatography-Flame Ionization Detector (GC-FID)-headspace technique has been applied to measure the odor intensity and character of perfume ingredients on impregnated textiles in the dry cleaning and wear cycle tests [13]. Similarly, the High Performance Liquid Chromatography (LC) has also been utilized to study the vanillin release property from Polysulfone/vanillin microcapsules in both hard water and pure water [14]. 

Apparently, the volatilization property of the core material of microcapsules also could be an entry point to study their release behavior. Sansukcharearnpon et al. [15] studied the fragrance release profile of microcapsules and fragrance core materials with TGA and electronic nose techniques. Based on the weight loss properties, a release curve could be obtained. Yeh et al. used a mathematical method to set up a release model of microcapsules containing a single core [16] and multi-cores [17]. Moreover, the spectral characteristics of core materials can also be utilized to analyze their release properties. For example, Ruben et al. [18] observed the crushing and residual property of prepared fluorescent dye microcapsules in finished fabric with a fluorescence microscope, and Bezerra et al. [19] evaluated the controlled-release mechanism of citronella oil microcapsules in different substrates with an attenuated total reflection FTIR spectrometer.

In the past few decades, the release behavior mechanisms, and models have been focused on the field of drug delivery [20,21]; however, the research has been very inadequate in the field of fragrance microcapsules. Although many of the reported works demonstrated very high theory and application values, there are still some major research challenges that need to be resolved, such as qualitative and quantitative analysis during the release process, the comparison of sustained and broken release characteristics, specific active core substance calibration, release-tracking studies, etc. 

In this paper, on the basis of our previous works [22,23], we fabricate fragrance microcapsules in a non-ionic system and study their sustained release and broken release behavior. The release profile of the prepared microcapsules is studied over a long time (2400 h) with a weighing method, and for the first time, we introduce the Solid Phase Microextraction-Gas Chromatography-Mass Spectrometer (SPME-GC-MS) to qualitatively and quantitatively analyze the sustained and broken release behavior of prepared microcapsules in finished fabrics.

## 2. Materials and Methods 

### 2.1. Materials

The core material is popular, softening essence oil (Rose® 7289, Double Horse Flavor and Fragrance Trade Co., Ltd., Tianjin, China), and the shell material is the pre-polymer of methanol-modified melamine-formaldehyde (MMF, solid content 78.0%, Honisite Chemical Trade Co., Ltd., Tianjin, China). The C_13_ isomeric alcohol ethoxylate (lutensol TO-7, BASF SE, Ludwigshafen, Germany) was used as the nonionic surfactant. The protective colloid is polyvinyl alcohol (PVA-1799) purchased from Letai Chemical Co., Ltd. (Tianjin, China). Acetic acid and sodium hydroxide (NaOH) was the analytical reagent and was supplied by Tianjin Kermel Chemical Reagent Development Center (Tianjin, China). The properties of the fabric used in this study are shown in Table 1. 

### 2.2. Preparation of MMF Microcapsules

PVA-1799 was dissolved in deionized water at 95 °C to give a 1% (w/w) aqueous solution. Under 45 °C, essence oil (20 g), TO-7 (1 g), PVA-1799 aqueous solution (5 g), and deionized water (57 g) were mechanically emulsified under a 10,000-rpm stirring rate for 5 min using a high-speed shearing emulsifying machine. Then, the obtained emulsion was poured into a 250-mL four-neck round-bottomed flask equipped with a reflux condenser and a Polytetrafluoroethylene anchor stirrer. The MMF pre-polymer (8.5 g) was dispersed in deionized water (6.5 g) and added into the reaction mixture under a mechanical stirring rate of 400 rpm and 45 °C. The emulsion pH value, determined by precision test paper and pH meter, was adjusted to 4 by adding 50.0% (w/w) acetic acid aqueous solution dropwise, and then, the reaction temperature was slowly and evenly increased to 75 °C over 60 min. After polymerization for 90 min, the reaction solution pH was adjusted to 7 by adding 30.0% (w/w) NaOH aqueous solution dropwise and then cooled down to room temperature to obtain the fragrance microcapsule suspension.

### 2.3. Impregnation of Microcapsules on Fabric Substrate

Firstly, the prepared fragrance microcapsule suspension (2 g) and deionized water (198 g) were mixed in a 500 mL glass beaker to give a 1.0% (w/w) fabric finishing agent. Then, the fabric substrates (6 mm × 18 mm) were placed into the solution and impregnated for 10 min under 25 °C. Eventually, the fabric sample was taken out and completely dried in an air-circulating oven at 50 °C. In addition, another fabric sample was impregnated with the same method using a mixture of essence oil (1 g), emulsifier Tween-80 (1 g), and deionized water (198 g) as a finishing agent. Then, the release performance of two fabric samples were tested and compared.

### 2.4. Chemical Structure and Morphology Characterization

The microcapsule suspension was filtered, washed with deionized water 3 times, and dried for 12 h in a vacuum oven at 50 °C to obtain solid samples without any free water or essence oil. Then, the pure solid samples were fabricated as KBr pellets and analyzed by a Thermo Nicolet 380 FTIR spectrometer (Thermo Fisher Scientific, Waltham, Massachusetts, USA), and the FTIR spectra were recorded with transmittance mode in the wavenumbers range of 400–4000 cm^−1^ (solution 4 cm^−1^). 

The morphology of the prepared microcapsule was characterized by optical microscope morphology (OM) and scanning electron microscope (SEM). The microcapsule suspension samples were dropped onto the surface of a glass slide and dried for 10 min under 50 °C. Then, the obtained samples and impregnated fabric samples mentioned in Section 2.3 were observed on an optical microscope (B203, Chongqing Optec Instrument Co., Ltd., Chongqing, China). For the SEM observation, the solid samples were sputtered with Au-conductive coating for 60 s and then observed with the SEM (JSM-7800F, Japan Electron Optics Laboratory JEOL Co., Ltd., Tokyo, Japan). 

The average particle size of the prepared microcapsules was measured by a laser particle size distribution analyzer (LS-POP III, Zhuhai OMEC instrument Co., Ltd., Zhuhai, China). The average particle size was characterized with *D_50_*, and the particle size distribution was presented by the ratio *P*, which was calculated by Equation (1):

(1) $$P=\frac{D_{\left(4,3\right)}}{D_{\left(3,2\right)}}$$

where *D*_(4, 3)_ is the volume mean diameter and *D*_(3, 2)_ is the area average diameter.

### 2.5. Odor Evaluation

The qualitative and quantitative odor release performance of the prepared fragrance microcapsule in the impregnated fabric samples (Table 2) were analyzed by SPME-GC-MS. The whole procedure was conducted as follows: The impregnated fabric samples (described in Section 2.3) were placed in a 4 mL vial (SUPELCO, 27136) and pre-treated with the different methods listed in Table 1. After equilibration at 25 °C for 5 min, the SPME manual extraction head (SUPELCO, 57330-U and 57324-U, 75um CAR/PDMS) activated at 250 °C for 30 min, was inserted into the vial for absorption, and then plugged into the GC inlet at 250 °C for 1 min. 

The GC-MS system (Agilent Technologies Inc., Santa Clara, California, USA) was the Network GC System 6890N and Mass Selective Detector 5975 equipped with HP-5 MS column (60 m × 0.25 mm, 0.25 μm), and the running system was controlled by MSD Chemstation. The carrier gas was helium (Purity 99.999%) at a 1 mL/min constant flow rate and a 10:1 split ratio. The oven temperature program was isothermal (50 °C) over 4.5 min, increased from 50 to 260 °C at a rate of 3 °C/min, and then increased to 270 °C at a rate of 10 °C/min and held isothermal for 10 min. The MS was operated in the electron impact ionization mode (70 eV), and the interface, source, and quadrupole temperatures were 280, 230, and 150 °C, respectively. In addition, the mass range was varied from 33 to 320 m/z. 

In order to identify the compounds in the samples, the resulting spectra were analyzed by manual spectral analysis based on NIST11 and a self-built library. The quantification of volatile compounds was analyzed and compared with GC peak height. 

### 2.6. Sustained Release Performance Evaluation

Some of the fragrance microcapsule suspension was poured into a petri dish (φ = 8 cm; weight *M_0_* = 7.0272 g) and quickly spread to form a semi-transparent film; its weight was recorded as *M_1_* (9.7375 g). Then, the petri dish was placed in the air-circulating oven and dried at 50 °C for 24 h to eliminate all the free water and un-encapsulated core material. Its weight was recorded as *M_2_* (7.5440 g). The drying process was conducted at 25 °C to simulate the conditions of dynamic release at normal temperature. The weight of the petri dish was weighed at 1-day intervals and recorded as *M_n_*. The mass of essence oil in the solid microcapsule samples was calculated by Equation (2), and the percentages of remaining essence oil (%) in the microcapsule samples during sustained release process were calculated by Equation (3). Thereby, a release curve of prepared microcapsules was obtained.

(2) $$M_{core}=\left(M_{1}-M_{0}\right)\left(\alpha +\beta \right)-(M_{1}-M_{2})$$

(3) $$W\%=\frac{M_{core}-(M_{2}-M_{n})}{M_{core}}\times 100\%$$

where *α* = 0.20 represented the mass fraction of the core material in the microcapsule samples and *β* ≈ 0.719 represented the mass fraction of the volatile component (except the core material) in the microcapsule samples. These two parameters were calculated based on the used raw material in Section 2.2.

## 3. Results and Discussion

### 3.1. Characterization of Microcapsules

**Figure 1:**
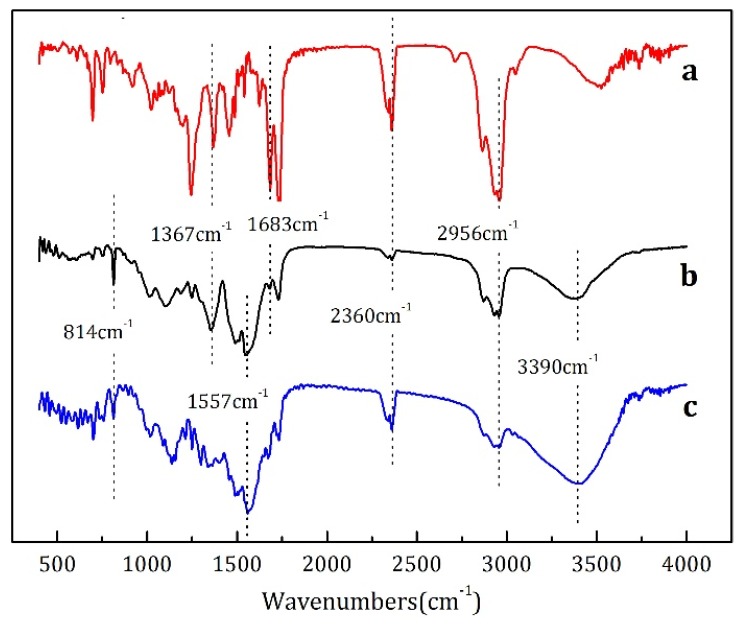
FTIR spectra of (**a**) essence oil, (**b**) microcapsules, and (**c**) methanol-modified melamine-formaldehyde (MMF) resin.

Figure 1 presents the FTIR spectra of (a) the essence oil, (b) solid microcapsules sample, and (c) MMF resin. It can be seen in curve a that the peaks at 2956 and 1367 cm^−1^ were caused by C–H stretching vibration and deformation vibration adsorption, respectively, and the split peak at 1683 cm^−1^ corresponded to the C=O stretching vibration adsorption peak. For MMF resin curve c, the strong and wide absorption peak at approximately 3390 cm^−1^ was attributed to the superposition of O–H and N–H stretching vibrations adsorption peak, and the peak at 2360 cm^−1^ illustrated that the MMF resin still had some unreacted –CN groups. The peaks at 1557 and 814 cm^−1^ belonged to the bending vibrations adsorption peaks of the triazine ring, which are the characteristic peaks of MF-based resins. For the characteristic peaks of the solid microcapsules sample (without free water and essence oil) shown in curve b, adsorption peaks attributed to both the MMF and essence oil were observed, suggesting that the solid microcapsule sample released essence oil during the test sample preparation process. Thus, it could be concluded that in the prepared samples, the essence oil was encapsulated inside the melamine resin.

**Figure 2:**
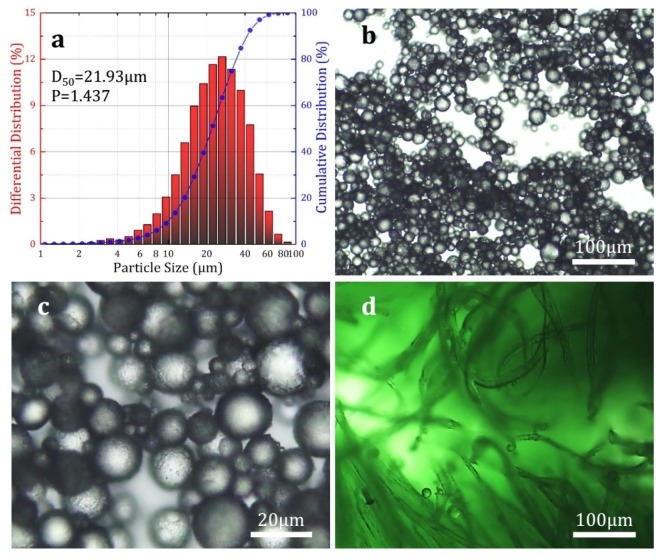
(**a**) Particle size and distribution of the prepared microcapsules, (**b**,**c**) microscopic morphology of the microcapsules, and (**d**) impregnated fabric samples.

The particle size analysis results of the prepared microcapsules, and the optical microscope observation results of the prepared microcapsules and impregnated fabric samples are shown in Figure 2. As shown in Figure 2a, the average particle size *D_50_* of the prepared microcapsules was 21.93 μm, and their distribution coefficient *P* was 1.437. It can be seen in Figure 2b,c that the morphology of the prepared microcapsule samples was a regular spherical shape with almost no surface damage, and they could be impregnated into the fabric substrate. Additionally, it was observed that the microcapsules were attached and embedded onto the surface and gap of the textile fibers (Figure 2d), which would make the fabric substrate show new odor release characteristics.

### 3.2. Odor Release Behavior of Prepared Microcapsules

In most situations, when the essence oil is directly released in the air, it shows a common diffusion mass transfer and follows Fick’s law. However, with respect to the fragrance microcapsules, as the core material (essence oil) was protected by the shell material and its release from inside the core to the outside environment was controlled, the release rate slowed down, and the release process was more complicated. Generally, the fragrance microcapsule release forms are classified into sustained release and broken release.

**Figure 3:**
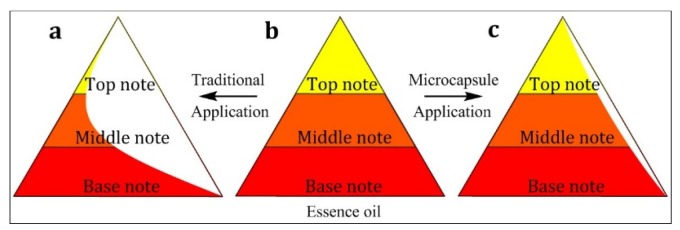
Aroma pyramid structure: (**a**) essence oil with traditional application, (**b**) newly produced essence oil, and (**c**) microencapsulated essence oil with different applications.

According to Poucher’s theory [24,25], the scent of perfume is divided into three parts: top note, middle note, and base note (Figure 3), and they describe the scent type and intensity of the aroma compositions volatilized at different times. In this work, we selected nine characteristic substances with different notes (Table 3) to study the odor release behavior of the different samples.

#### 3.2.1. Sustained Release

**Figure 4:**
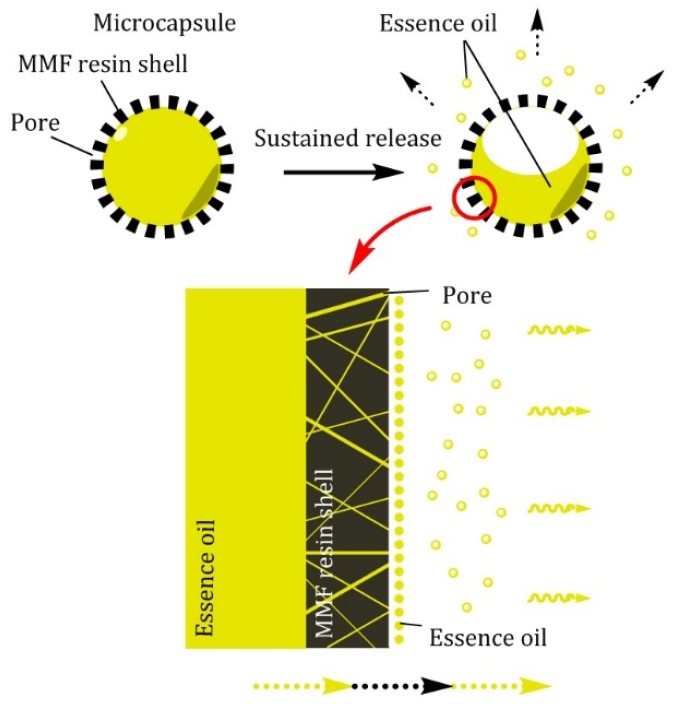
Schematic of the sustained release of essence oil from the prepared fragrance microcapsules.

**Figure 5:**
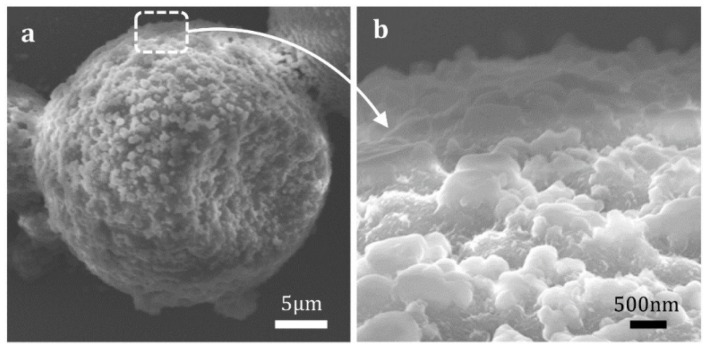
SEM images of the prepared microcapsule (**a**) and its surface morphology (**b**).

For the sustained release of essence oil from the prepared microcapsules, it was considered that the essence oil firstly permeated from the inside core to the shell material, further migrated to the surface, and then showed similar volatilization and diffusion states to those of liquid essence oil under normal circumstances (Figure 4). However, it should be noted that the essence oil release from the microcapsules was restricted by the surface morphology and porosity of microcapsules [16,26]. The surface morphology of the prepared microcapsules was analyzed by SEM, and the results are shown in Figure 5. It was found that the prepared microcapsules had very rough surface structures, and it made them possess large surface areas. Additionally, the special surface structure was beneficial to the volatilization of the essence oil migrating from the inside core of the microcapsules. Meanwhile, the migration rate of the core material essence oil to the outside shell surface was slow, and thus, from the viewpoint of dynamics, the sustained release rate of the essence oil from the microcapsules was mainly determined by its migration rate in the shell material. 

**Figure 6:**
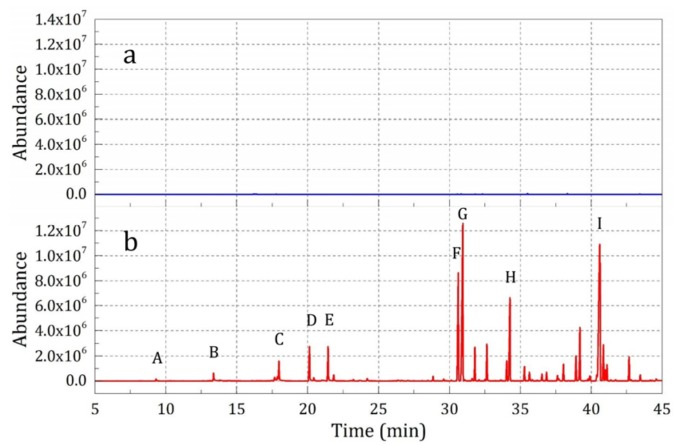
SPME-GC-MS analysis results of the samples with different absorption times: (**a**) 10 min (Sample 1), and (**b**) 12 h (Sample 2)

The release properties of essence oil from the microcapsules over a short time (10 min, Sample 1) and a long time (12 h, Sample 2) were tested, and the results are shown in Figure 6. It was found that over a short time, negligible essence oil was detected (Figure 6a); however, over a long time a series of essence oil compounds were detected obviously (Figure 6b).

By comparing with the database, the peak intensity of the selected substrates was shown in Table 4. For Sample 1, almost no top note substrates were detected, and only the middle note substrate 1, 8-cineole and some base note substrates were detected, suggesting that the fabric samples finished with the prepared microcapsules had a very faint scent. However, as shown in Sample 2, all the note substrates were detected after a long release time, which illustrated that the prepared microcapsules had a seal protection effect and controlled-release performance on the core material.

**Figure 7:**
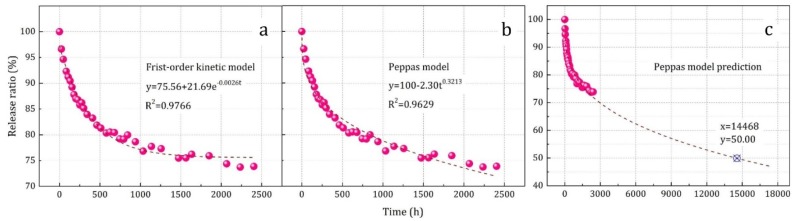
The release ratio data and fitting results of the prepared microcapsule core material during sustained release: (**a**) First-order kinetic model, (**b**) Peppas model, (**c**) Peppas model prediction.

The seal protection effect and controlled-release performance could be analyzed by SPME-GC-MS technology, especially transient or short-term changes (such as at initial time t_0_ or at a specific time t_n_), but it is difficult to describe the dynamic process. Therefore, in order to investigate the controlled-release performance of the prepared microcapsules, a weighing calculation method was used to test the release rate of essence oil from the prepared microcapsules over 2400 h, and the results are shown in Figure 7. It was found that the mass percentage of the essence oil continuously decreased, and the decrease rate gradually became slower as the release time increased. After fitting and comparing with various kinetic models, it was found that the release rate was consistent with both the first-order kinetic (Figure 7a) and the Peppas model (Figure 7b), and they were in accordance with Equation (4) as follows:

*y = a + b·e^−k·t^* (first-order kinetic model) Result: y = 75.56 + 21.69·e^−0.0026t^ (R^2^ = 0.9766). (4)

*y* = 100 − *a·t^k^* (Peppas model) Result: y = 100 − 2.30t^0.3213^ (R^2^ = 0.9629). (5)

According to the fitting results, the R^2^ of the first-order kinetic model was higher, indicating that this model was more correlated with the experimental data. At the same time, the parameter *a* = 75.56 in Equation (4) represented the release limitation of 75.56%, meaning that the microcapsules can only release 24.44% of the total essence oil. However, in the experiment, it was found that after 2400 h of sustained release, the microcapsule samples still showed a light but distinct odor, which suggested that the release limitation might be lower than the fitting parameter *a*.

In view of this point and the overall trend of the weight loss rate, the Peppas model might be more suitable for the further description of release dynamic processes, and the parameter *k* = 0.3213 indicated that the sustained release of the prepared microcapsules basically followed the Fickian diffusion character. Furthermore, the sustained release half-life was predicted under the Peppas model, and the results are shown in Figure 7c. The calculated result was 1.65 years, and it implied that the prepared microcapsules possessed a long application and storage time.

#### 3.2.2. Broken Release

**Figure 8:**
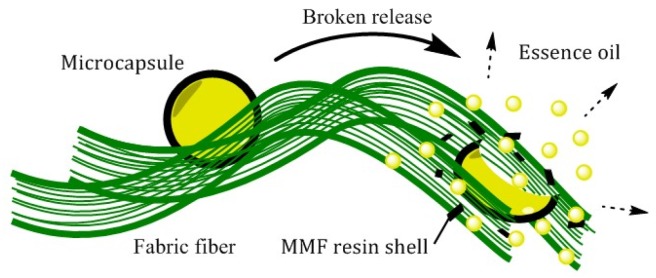
Schematic of the broken release of fragrance microcapsules on the fabric under external force.

Under natural situations, the prepared microcapsules showed a sustained release state. But when external forces acted on the finished fabric, the contained microcapsules were broken, and the essence oil would quickly release to show a broken release property; the schematic is shown in Figure 8. 

**Figure 9:**
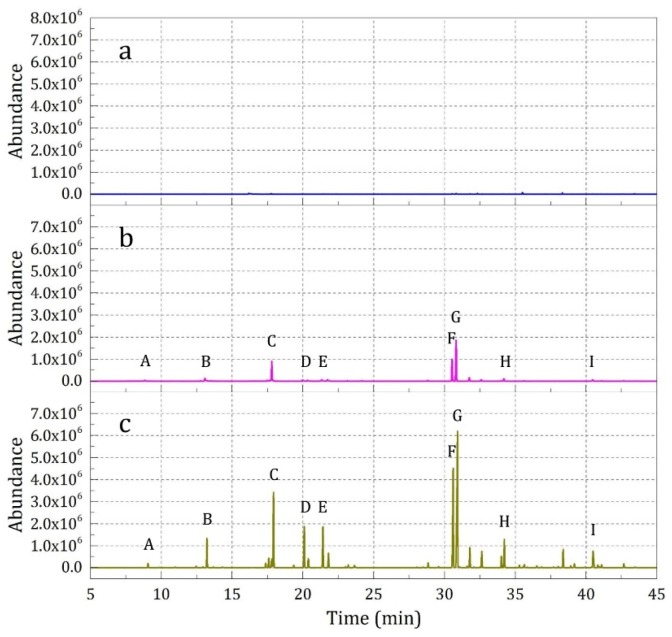
SPME-GC-MS analysis results of the samples under different external force: (**a**) no hitting (Sample 1), (**b**) hitting 1 time (Sample 3), and (**c**) hitting 5 times (Sample 4).

Figure 9 and Table 5 present the SPME-GC-MS analysis results of the released essence oil composition from the finished fabric under a natural state and external force (Samples 1, 3 and 4). It was found that the species and relative intensities of the characteristic peaks of the released essence oil composition were very different before and after external hitting, and the hitting indeed made the microcapsules show broken release properties, as expected. Meanwhile, the hitting times showed a significant effect on the release amount of the essence oil. However, due to the spatial distribution of the microcapsules on the fabric, the hitting effect for the fabric samples was uneven. In addition, the fabric itself had a certain inhibiting, absorbing, and releasing effect; thus the characteristic peak intensity for the samples hitting for 1 time and 5 times ranged from 3.18 to 35.54 multiples (curves b and c).

#### 3.2.3. Sustained–Broken Release Comparison

(1) Broken Release Properties after Different Sustained Release Times

**Figure 10:**
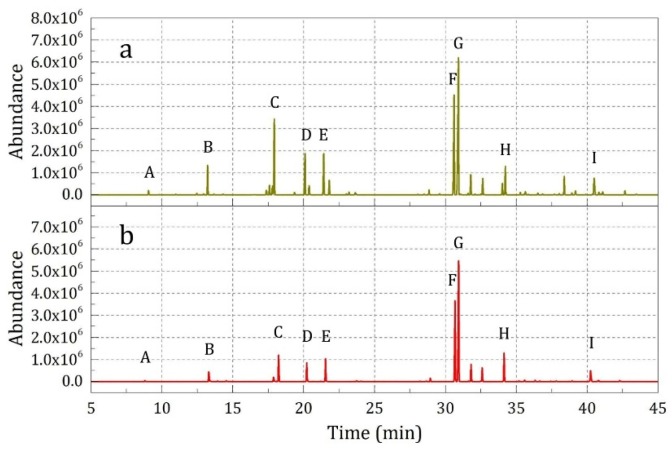
SPME-GC-MS analysis results of the samples under external force after sustained release for different times: (**a**) 0 h (Sample 4) and (**b**) 2400 h (Sample 7).

In order to further investigate the airtightness and broken release property of the prepared microcapsules, the essence oil composition released from the impregnated fabric samples with and without 2400 h sustained release and further hitting o 5 times (Samples 4 and 7) was analyzed, and the results are shown in Figure 10 and Table 6 for the impregnated fabric samples. It could be seen that although Sample 7 had undergone a 2400 h sustained release time, Sample 4 without sustained release showed only a 1.02–3.78 times higher peak intensity. The obtained experimental results further verified that the prepared microcapsules showed a very slow sustained release rate and maintained good broken release properties under external force even after a long, sustained release time.

Additionally, it was found that the essence oil compositions in different notes showed different release rates. In the top note, the ratio of the characteristic peak intensities between Sample 4 and Sample 7 was 3.01–3.78. However, in the base note, this ratio was only 1.02–1.57. The difference suggested that for the essence oil compositions inside the microcapsule core, their volatile characteristics could also affect the release rate, and the higher volatility of the substances, the greater decrease in the release rate. 

Moreover, Sample 7 showed good broken release properties under external force after sustained release for 2400 h, and the top note and middle note substances had a large amount of residue. This provided some support to the model analysis results shown in Figure 7 and, indeed, illustrated that the release limitation was far more than the fitting parameter *a* in Equation (4).

(2) Comparison between Sustained Release and Broken Release

**Figure 11:**
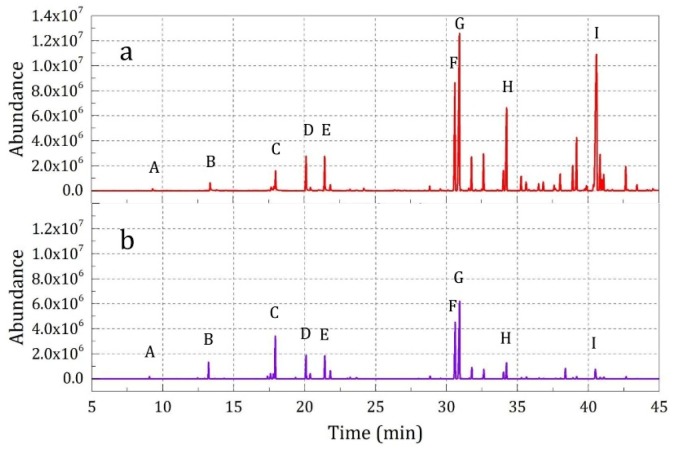
SPME-GC-MS analysis results of the samples in the sustained release state (**a**) (Sample 2) and the broken release state (**b**) (Sample 4).

The different release properties of the essence oil from the impregnated fabric samples in the sustained release state (Sample 2) and broken release state (Sample 4) were also investigated, and the results are shown in Figure 11 and Table 7. It can be seen that in different release states, the samples showed obviously different behaviors. The characteristic peak intensity for composition A–C in curve a (sustained release) was lower than that in curve b (broken release); however, the characteristic peak intensity E–I showed the opposite trend. The results illustrate that the middle and base note odor showed advantages in the sustained release state, and the top note odor was more outstanding in the broken release state. This conclusion also could be verified by the data comparison list in Table 7.

#### 3.2.4. Release Property of Fabrics Finished by Essence Oil and Microcapsules

**Figure 12:**
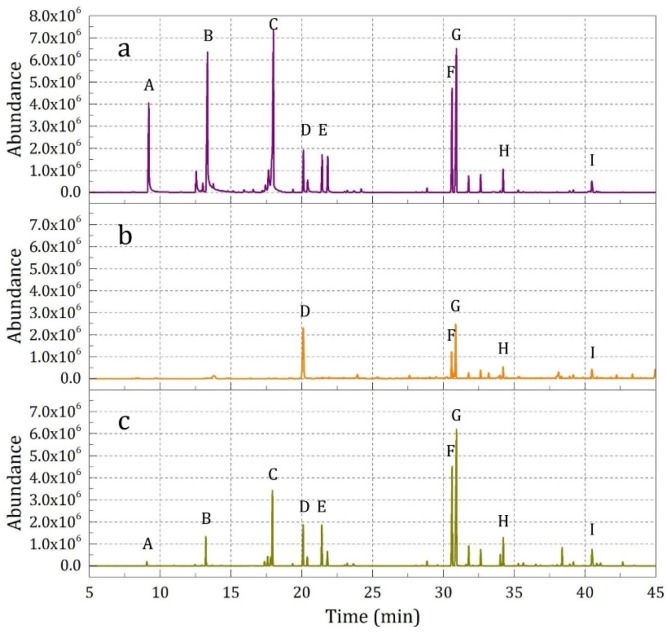
SPME-GC-MS analysis results of the samples: (**a**) 5 drops of essence oil (Sample 6), (**b**) fabric finished with the essence oil (Sample 5), and (**c**) fabric finished with the fragrance microcapsules (Sample 4).

The essence oil compositions released from the pure essence oil (Sample 6), fabric samples finished with essence oil (Sample 5), and those finished with microcapsule suspension (Sample 4) were analyzed by SPME-GC-MS. The results are shown in Figure 12. By comparison with the pure essence oil (curve c), it was found that in the fabric sample finished with the essence oil suspension (curve b), the characteristic peak species, numbers, and intensities decreased, which could be seen from the c/b ratio shown in Table 8. This change illustrated that in the fabric samples finished with pure essence oil, the top note odor almost vanished and the middle note lost potency remarkably, which resulted in significant difference between the released odor and the designed odor (such as in Figure 3a). The reason for this was that the fabric could not reconcile the essence oil mixture due to their different physicochemical properties, such as solubility, polarity, and characteristic groups. Furthermore, the finishing and drying process resulted in a certain loss of and chemical changes in the essence oil. By comparison, in the fabric sample finished with the prepared microcapsule suspension (curve c), the characteristic peak species and intensities were similar to those of the pure essence oil, suggesting that the prepared microcapsule showed an excellent odor recovery and explosiveness (such as in Figure 3c). In particular, most of the middle note and base note essence oil compositions were the same as those of the pure essence oil, which could be expressed via the c/a ratio shown in Table 8.

## 4. Conclusions

The fragrance microcapsules were fabricated in a non-ionic system, and series of characterization results showed that the essence oil was encapsulated inside the melamine resin. The sustained release properties of the prepared microcapsules were analyzed with a weight calculation method over a release time of 2400 h, and the results showed that the essence oil weight in the microcapsules decrease continuously. Moreover, according to the mathematical fitting, the release trend was consistent with a Peppas model (y = 100 − 2.30t^0.3213^, R^2^ = 0.9629). 

The sustained and broken release properties of the microcapsules in the impregnated fabric samples was qualitatively and quantitatively analyzed by SPME-GC-MS method, and the results showed that in different release states, the release essence oil from the fabric samples showed different note properties. In the sustained release state, the middle and base note odor showed advantages; however, in the broken release state, the top note odor was more outstanding. Furthermore, it was found that the characteristic peak species and intensities of the sample finished with the prepared microcapsule were very similar to those of the pure essence oil, suggesting that the prepared microcapsule showed an excellent property of odor recovery and strength. 

In conclusion, the work proposes a new qualitative and quantitative analysis method for the field of fragrance microcapsules, and the SPME-GC-MS technology might provide a suggested analysis strategy for other controlled-release materials and the surface/interface mass transfer phenomenon. 

## Figures and Tables

**Table 1:** Properties of fabric substrate.

Style	Weave	Weight (g·m^−2^)	Thickness (mm)	width (mm)	Length (mm)
100% Cotton	Plain	156	0.4	6	18

**Table 2:** Different pre-treated methods on the impregnated fabric samples.

Sample	Substrate	Finishing Agent	Treatment	Absorption Time
1	Fabric	Microcapsule	No hitting^1^	10 min
2	Fabric	Microcapsule	No hitting	12 h
3	Fabric	Microcapsule	Hitting 1 time	10 min
4	Fabric	Microcapsule	Hitting 5 times	10 min
5	Fabric	Essence oil + emulsifier	No hitting	10 min
6	5 drops essence oil	Null	No hitting	10 min
7	Fabric	Microcapsule	Standing for 2400 h at 25 °C, hitting 5 times	10 min

^1^ Hitting treatment: A Φ = 5 mm, L = 20 mm glass rod was used to hit the fabric samples (break microcapsules) in the vial with free fall at the height of 4 cm for different times, and the cap was quickly sealed.

**Table 3:** Selected substances with different volatilization times and different notes.

Notes	Peaks	Substances
Top note	A	Ethyl-2-Methylbutyrate
	B	Ethy2-Methylvalerate
Middle note	C	1,8-Cineole
	D	Dihydromyrcenol
	E	Tetrahydrolinalool
Base note	F	Isobornyl acetate
	G	4-tert-Butylcyclohexyl acetate
	H	3,5,5-Trimethylhexyl Acetate
	I	Tricyclodecenyl propionate

**Table 4:** The chromatographic peak intensities and ratios of the samples with different adsorption times.

Peaks	Samples		b/a
	(a) Sample 1	(b) Sample 2	
A	—	141935	—
B	—	579547	—
C	22765	1583204	69.55
D	—	2803734	—
E	—	2759339	—
F	16756	8256025	492.72
G	33312	11431463	343.16
H	—	6143374	—
I	9979	10876780	1089.97

**Table 5:** The intensity and ratios of the chromatographic peaks of samples with different hitting times.

Peaks	Samples			c/b
	(a) Sample 1	(b) Sample 3	(c) Sample 4	
A	—	30239	183224	6.06
B	—	123145	1325714	10.77
C	22765	898194	3501869	3.90
D	—	51882	1844140	35.54
E	—	64510	1808735	28.04
F	16756	992325	4448527	4.48
G	33312	1843702	5872087	3.18
H	—	111938	1294174	11.56
I	9979	60428	754148	12.48

**Table 6:** The chromatographic peak intensity and ratios between the samples under external force after sustained release for different times.

Peaks	Samples		a/b
	(a) Sample 4	(b) Sample 7	
A	183224	48438	3.78
B	1325714	440152	3.01
C	3501869	1187322	2.95
D	1844140	843829	2.19
E	1808735	1027600	1.76
F	4448527	3641310	1.22
G	5872087	5425702	1.08
H	1294174	1271108	1.02
I	754148	481820	1.57

**Table 7:** The chromatographic peak intensities and ratios between the samples in the sustained release state and the broken release state.

Peak	Samples		b/a
	(a) Sample 2	(b) Sample 4	
A	141935	183224	1.29
B	579547	1325714	2.29
C	1583204	3501869	2.21
D	2803734	1844140	0.66
E	2759339	1808735	0.66
F	8256025	4448527	0.54
G	11431463	5872087	0.51
H	6143374	1294174	0.21
I	10876780	754148	0.07

**Table 8:** The chromatographic peak intensities and ratios of different samples.

Peaks	Samples			c/a	b/a
	(a) Sample 6	(b) Sample 5	(c) Sample 6		
A	4042352	—	183224	0.05	—
B	5783880	—	1325714	0.23	—
C	6839605	—	3501869	0.51	—
D	1804020	2286585	1844140	1.02	1.27
E	1619746	—	1808735	1.12	—
F	4469289	1167465	4448527	1.00	0.26
G	5931929	2308449	5872087	0.99	0.39
H	1050035	513144	1294174	1.23	0.49
I	483660	374041	754148	1.56	0.77
